# Arthroscopic minimum saucerization and inferior-leaf meniscectomy for a horizontal tear of a complete discoid lateral meniscus: Report of two cases

**DOI:** 10.1016/j.ijscr.2018.11.027

**Published:** 2018-11-22

**Authors:** Akira Tsujii, Tomohiko Matsuo, Kazutaka Kinugasa, Yasukazu Yonetani, Masayuki Hamada

**Affiliations:** a Department of Orthopaedic Surgery, Hoshigaoka Medical Center, 4-8-1, Hoshigaoka, Hirakata, Osaka, 573-8511, Japan; b Department of Orthopaedic Surgery, Moriguchi Keijinkai Hospital, 2-47-12, Yagumohigashimachi, Moriguchi, Osaka, 570-0021, Japan; c Department of Sports Orthopaedics, Osaka Rosai Hospital, 1179-3, Nakagasone-cho, Kita-ku, Sakai, Osaka, 591-8025, Japan

**Keywords:** Discoid lateral meniscus, Horizontal tear, Saucerization, Inferior-leaf meniscectomy

## Abstract

**Introduction:**

Treatment of a horizontal tear of a complete discoid lateral meniscus (DLM) is still controversial. Preserving peripheral rim as a normal shape of the meniscus with single-leaf resection is a conventional treatment, however meniscal function could not be fully restored.

**Presentation of case:**

A 28-year old woman and a 34-year old woman experienced knee pain and had restricted knee extension. MRI showed horizontal tears of complete DLM in both patients. Arthroscopic minimum saucerization preserving more than 10 mm peripheral rim and inferior-leaf meniscectomy was performed. Two years after the surgery, the patient had no pain and no restriction of ROM. MRI showed the remaining superior-leaf maintained about half its width and no progression of coronal/sagittal extrusion.

**Discussion and conclusion:**

As resecting more meniscal tissue has been considered to be a cause of degeneration or extrusion of the meniscus, arthroscopic minimum saucerization, preserving more meniscal tissue than standard saucerization, and inferior-leaf meniscectomy can be an alternative treatment option of horizontal tears of complete DLM with satisfying clinical and radiological results.

## Highlights

• Arthroscopic minimum saucerization and inferior-leaf meniscectomy at 2-year follow-up.
• Preserving more than 10 mm width could obtain excellent clinical outcomes.
• Leaving more meniscal tissue might prevent meniscal extrusion.

## 1 Introduction

A horizontal tear is the most common tear pattern of a complete discoid lateral meniscus (DLM) [1,2]. Previous studies showed that a horizontal tear including a complex tear occurred in 55–71% of complete DLM tears [[1], [2], [3]]. Because of its abnormal structure and heterogeneously arranged fibers, DLM is considered to be vulnerable [[4], [5], [6]].

Conventionally, symptomatic DLM tear is treated by removing only the central portion and preserving 6–8 mm peripheral rim to restore normal shape of the meniscus (known as ‘saucerization’) with or without repair, and successful clinical outcomes have been reported [7,8]. And a horizontal tear of the DLM is also treated by saucerization and resecting both leaves or single-leaf [[9], [10], [11]]. However, even preserving peripheral rim as a normal meniscus could not prevent meniscal extrusion or degenerative changes [8,[12], [13], [14], [15]].

Thus, preserving more meniscal tissue might be necessary to restore meniscal function. However, there have been no reports on how much width could be preserved without any symptoms, regarding a horizontal tear of DLM. We are now preserving more than 10 mm peripheral rim with inferior-leaf meniscectomy. And here, we present two cases of arthroscopic minimum saucerization and inferior-leaf meniscectomy and the satisfying clinical and radiological outcomes at 2 years postoperatively. Senior surgeon (M.H.), who had experience in arthroscopic surgery for more than 30 years, performed both operations. It is reported in line with the PROCESS criteria [16].

## 2 Presentation of case

### 2.1 Case 1

**Fig. 1:**
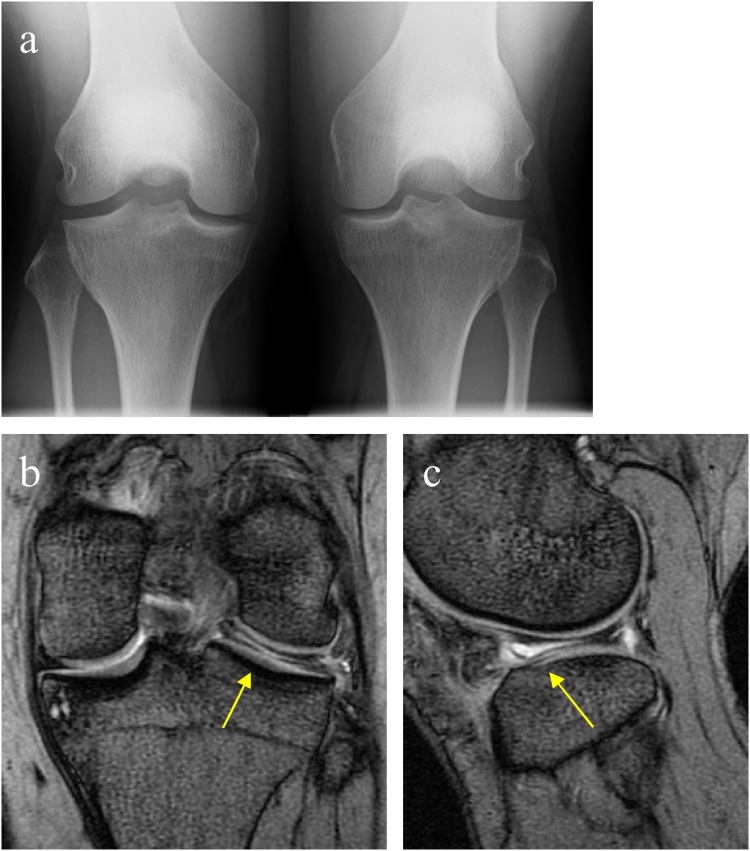
Preoperative images of the first case. Rosenberg-view radiograph of bilateral knees (a). T2-weighted MRI shows a discoid lateral meniscus with a horizontal high signal cleavage (arrow) throughout its body in mid-coronal (b) and mid-sagittal (c) sections.

A 28-year-old female, who was a childminder, injured her left knee during repeated deep flexion of the knee to comfort the children. She had a pain in the lateral side of the knee and standing-up was the most painful motion for more than 3 months. On physical examination, the knee was restricted in extension to 5° without instability, and the McMurray test [17] was positive. X-ray showed lateral joint space widening compared to the right knee (Fig. 1a). Magnetic resonance imaging (MRI) showed a horizontal high signal cleavage throughout the body of the DLM (Fig. 1b, c).

**Fig. 2:**
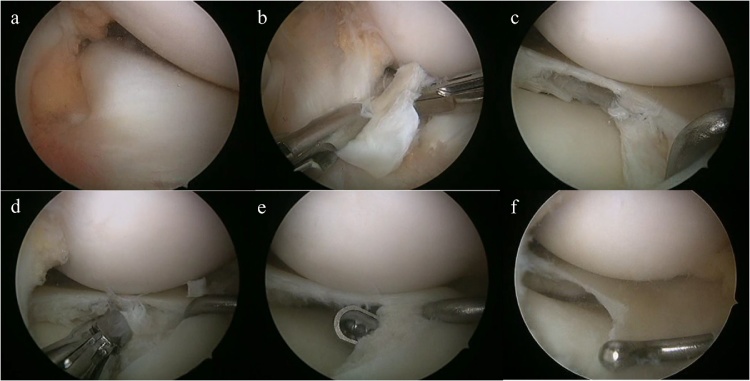
Intraoperative arthroscopic images of the first case. No tear on the femoral side of the discoid lateral meniscus (a). Resection of the central portion (b). Horizontal tear throughout its body (c). Resection of the inferior leaf with a forceps (d). Resection of the anterior part of the inferior leaf with a shaver via an inferomeniscal portal (e). About half of the width of the remaining stable leaf is preserved (f).

After finding that there was no tear on the femoral side of the DLM and confirming its stability by a probe using two standard anterior portals (Fig. 2a), a minimal part of the central portion of the DLM was removed using an additional far-anteromedial portal (Fig. 2b, c). Then, the middle and posterior part of the inferior-leaf was removed with a meniscal punch, and the anterior part was removed with a shaver using an inferomeniscal portal (Fig. 2d, e) [18]. About half of the width of the remaining stable superior-leaf was preserved (Fig. 2f, Video). Finally, the knee was arthroscopically checked its smooth flexion and extension without clicking.

Postoperatively, the patient had free knee range of motion (ROM) and weight bearing. All activities were allowed at 1 month postoperatively.

**Fig. 3:**
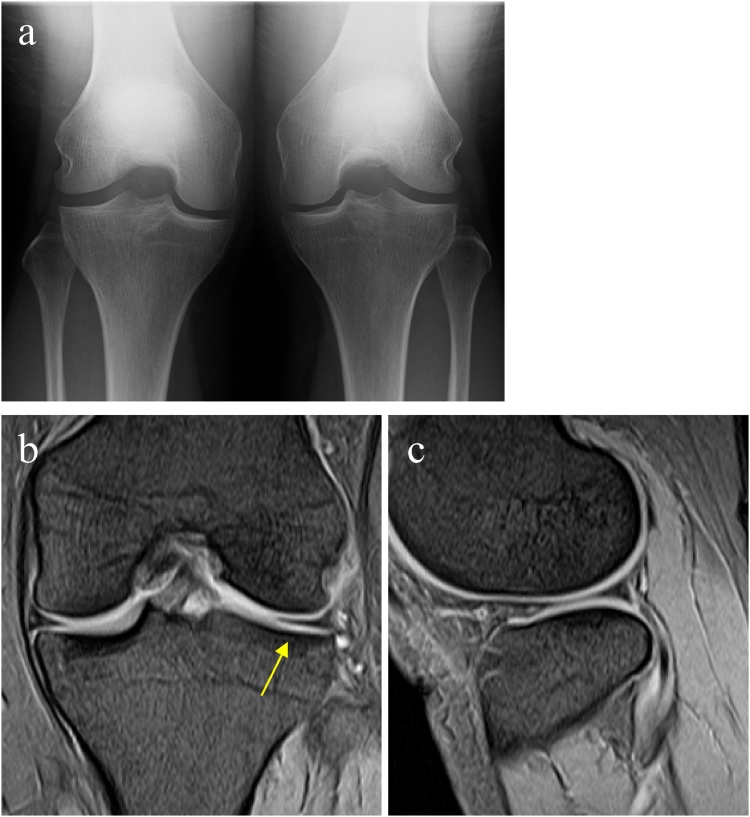
Postoperative images of the first case at 2 years after the surgery. Rosenberg-view (a). Mid-coronal (b) and mid-sagittal (c) T2-weighted MRI images.

Two years after the surgery, the patient had no pain and no restriction of ROM. X-ray showed slight narrowing of the lateral joint space, but no other degenerative changes (Fig. 3a). On MRI, the remaining superior-leaf maintained about half its width (14.0 mm) with no intrameniscal signal changes, and no progression of coronal/sagittal extrusion (Fig. 3b, c).

### 2.2 Case 2

**Fig. 4:**
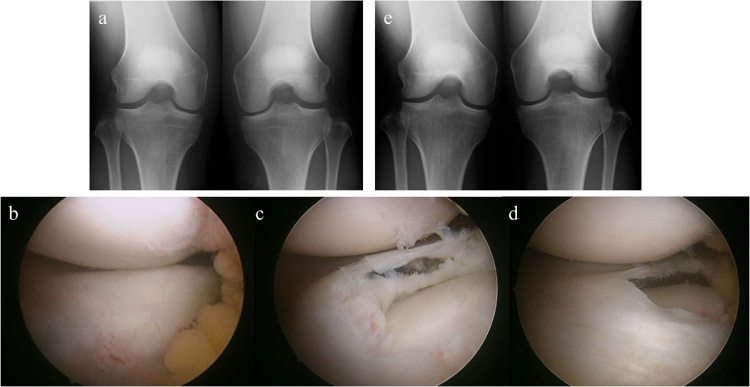
Images of the second case. Preoperative Rosenberg-view radiograph of bilateral knees (a). Intraoperative arthroscopic images; no tear on the femoral side of the discoid lateral meniscus (b), horizontal tear throughout its body (c), and about half of the width of the remaining stable leaf is preserved (d). Postoperative Rosenberg-view (e) at 2 years after surgery.

A 34-year-old female had felt pain in her right knee while running. She had a pain in the lateral side and running was the most painful motion for more than 3 months. On physical examination, knee extension was restricted to 10°. The McMurray test [17] was positive without instability. X-ray showed no remarkable findings (Fig. 4a). MRI showed that the DLM had a horizontal high signal cleavage. Surgery and postoperative therapy were performed as in the first case (Fig. 4b–d).

Two years after the surgery, the patient had no symptoms while running, and had no restriction of ROM. X-ray showed slight narrowing of the lateral joint space (Fig. 4e), and MRI showed a thin lateral meniscus that had maintained its width (12.1 mm) with no progression of coronal/sagittal extrusion.

## 3 Discussion

The key finding of the present study was that arthroscopic minimum saucerization and inferior-leaf meniscectomy for horizontal tear of complete DLM preserving approximately half-width of the superior leaf could obtain excellent clinical outcomes, and the meniscus exhibited no extrusion. This procedure is suggested to be an alternative treatment option of horizontal tears of complete DLM.

Previously, saucerization, resecting the central portion to create a normal shape (6–8 mm in width), with single-leaf partial meniscectomy has been reported as a treatment option for a horizontal tear of symptomatic DLM [10,11]. However, in both reports there was an underlying principle that removing the central portion, saucerization, should first be performed to create a normal shape of meniscus, and how much width could be preserved was not sufficiently discussed. Furthermore, arthroscopic images showed little meniscal tissue was left compared to the present cases [10]. Recently, it was reported that residual meniscal width less than 5 mm was shown to be a risk factor for degeneration in treating DLM tears [19]. Additionally, after saucerization with peripheral repair for DLM tears, postoperative extrusion was observed from 2 weeks to 6 months after surgery [8]. Thus, in the present cases, the aim was to preserve as much meniscal tissue as possible without regard to a normal meniscal shape in treating horizontal tears of symptomatic DLM, and consequently, meniscal width was maintained at more than 10 mm with satisfying clinical outcomes. In addition, postoperative MRI showed no progression of meniscal extrusion. Preserving more meniscal tissue could restore more collagen network and it might be effective to prevent its displacement. Long-term follow-up is necessary for early detection of degenerative changes.

When preserving as much volume of the superior-leaf as possible, resecting the anterior part of the inferior-leaf becomes harder because of poor visualization and a difficult approach to the inferior-leaf through standard portals. Previously reported, far-anteromedial portal could improve visualization [20], and an inferomeniscal portal could achieve direct access of the shaver to the inferior-leaf [18,21]. These additional portals are indispensable for success with this technique.

## 4 Conclusion

The combination of arthroscopic minimum saucerization and inferior-leaf meniscectomy can be a good surgical option for a horizontal tear of a complete discoid lateral meniscus.

## Conflict of interest

The authors declare no conflicts of interest.

## Funding source

This research did not receive any specific grant from funding agencies in the public commercial, or non-for-profit sectors.

## Ethical approval

The report of cases was approved by the ethical committee of the Hoshigaoka Medical Center 5th July, 2018 and the admission number is 1874.

## Consent

Written informed consent was obtained from the patients for publication of this case report and accompanying images. A copy of the written consent is available for review by the Editor-in-Chief of this journal on request.

## Author contribution

Akira Tsujii collected and analyzed data. Akira Tsujii and Masayuki Hamada wrote the manuscript. All authors collaborated in the patient’s medical care and approved the final article.

## Registration of research studies

Researchregistry4385.

## Guarantor

Masayuki Hamada.

## Provenance and peer review

Not commissioned, externally peer reviewed.

## Appendix A Supplementary data

Video 1 The technique of arthroscopic minimum saucerization and inferior-leaf meniscectomy.
